# Methodological considerations on the validation of the CuidaSM Scale

**DOI:** 10.11606/s1518-8787.2026060007752

**Published:** 2026-08-31

**Authors:** Arthur Brandão Siqueira Neves de Lima, Hugo Rafael de Souza e Silva

**Affiliations:** I Universidade de Pernambuco Faculdade de Ciências Médicas Recife PE Brasil Universidade de Pernambuco. Faculdade de Ciências Médicas. Recife, PE, Brasil

Dear Editor,

We hereby present methodological considerations regarding the article “Mental Health Care Need Assessment Scale: Additional Evidence,” by Mendonça et al.^1^ The urgency of this communication stems from the fact that CuidaSM was adopted by the Ministry of Health in the document “Implementation of the Mental Health Care Pathway in Primary Health Care for Network Organization,” known as Mental Health in Primary Health Care^2^. A news article on the IdeiaSUS Fiocruz website reports that “Nova ferramenta pode transformar a maneira como o Brasil lida com a saúde mental no SUS” (“New tool may transform the way Brazil addresses mental health in the SUS”), with nationwide implementation expected by mid-2026^3^. Methodological weaknesses in the validation study therefore have direct implications for the population served by this policy.

We note, first, an editorial concern: the original study of the instrument (reference 9 in the article by Mendonça et al.^1^) consists of a preprint on Research Square, without a definitive DOI and without a completed peer review. The publication of “additional” evidence from an instrument whose primary study has not yet been formally indexed is a situation that warrants consideration by this journal.

The most critical methodological problem lies in the dichotomous nature of all items on the scale, combined with the absence of a statement regarding the estimation method. For binary data, best practice requires: (1) a tetrachoric correlation matrix—rather than Pearson’s correlation, which produces distorted factor loadings in this context—; and (2) the WLSMV or ULSMV estimator, available in Jeffreys’s Amazing Statistics Program (JASP), the software used in the study itself. The omission of these choices prevents an assessment of whether the validity inferences are methodologically sound^4^.

Regarding the sampling design, the article does not present a sample size calculation and employs convenience sampling, with more than 58% of participants coming from the Southeast region. Given the stated objective of national validation, the absence of a measurement invariance analysis across regions constitutes a significant shortcoming. Additionally, samples exceeding 400 cases increase statistical power and may mask the effects of multivariate non-normality—an assumption that, just like the checking for multivariate outliers, this is not even mentioned in the article, thereby compromising the confirmatory factor analysis (CFA) regardless of the fit indices obtained.

The convergent validity of the model was also not adequately demonstrated. According to widely accepted rules of thumb, as illustrated in Hair et al.^4^, factor loadings (λ) ≥ 0.5, extracted average variance (EVA) ≥ 0.5, and construct reliability ≥ 0.7 would be required, at a minimum. None of these indicators are reported. The use of network analysis on the same sample as that used for exploratory factor analysis (EFA) and AFC does not constitute independent validation but merely offers another perspective on the same covariance patterns^5^, and does not compensate for the lack of cross-validation in an independent sample.

Finally, although the reliability measures (Cronbach’s alpha, GLB, and omega) show satisfactory values, high reliability does not constitute evidence of validity—a fundamental distinction that the article does not make explicit.

In light of the above, we request that the Editor: (1) publish this letter as a comment on the article, with the authors’ right of reply; and (2) evaluate the appropriateness of requesting an errata or addendum that specifies the estimator used, the assumptions verified, and the indicators of convergent validity. The methodological soundness of a nationwide mental health assessment tool is a responsibility that transcends academic debate.
