# Supplementary material for: Methodological considerations on the validation of the CuidaSM Scale
Source: Rev Saude Publica. 2026 Aug 31;60:e37. doi: 10.11606/s1518-8787.2026060007752 (PMC13645322; doi:10.11606/s1518-8787.2026060007752)
Supplement: Translation

# Translation: Considerações metodológicas sobre a validação da Escala CuidaSM

**Authors:** Arthur Brandão Siqueira Neves de Lima, Hugo Rafael de Souza e Silva

**Affiliations:** I Brasil Universidade de Pernambuco. Faculdade de Ciências Médicas. Recife, PE, Brasil

Ilustríssimo Sr. Editor,

Vimos, por meio desta, apresentar considerações metodológicas sobre o artigo “Escala de Avaliação da Necessidade de Cuidado em Saúde Mental: evidências adicionais”, de Mendonça et al.^1^A urgência desta comunicação decorre do fato de que a CuidaSM foi adotada pelo Ministério da Saúde no documento “Implementação da linha de cuidado de saúde mental na APS para organização da rede”, conhecido como Saúde Mental na APS^2^. Uma notícia no portal da IdeiaSUS Fiocruz divulga que “Nova ferramenta pode transformar a maneira como o Brasil lida com a saúde mental no SUS”, com implantação nacional prevista até meados de 2026^3^. Fragilidades metodológicas no estudo de validação trazem, portanto, implicações diretas para a população assistida por essa política.

Registramos, inicialmente, uma preocupação editorial: o estudo de origem do instrumento (referência 9 no artigo de Mendonça et al.^1^) consiste em um *preprint* no *Research Square* , sem DOI definitivo e sem revisão por pares concluída. A publicação de evidências “adicionais” de um instrumento cujo estudo primário ainda não foi formalmente indexado constitui uma situação que merece reflexão por parte deste periódico.

O problema metodológico mais crítico reside na natureza dicotômica de todos os itens da escala combinada à ausência de declaração do método de estimação. Para dados binários, a prática recomendada exige: (1) matriz de correlações tetracóricas – e não a correlação de Pearson, que produz cargas fatoriais distorcidas nesse contexto –; e (2) estimador WLSMV ou ULSMV, disponíveis no *Jeffreys’s Amazing Statistics* Program (JASP), *software* utilizado no próprio estudo. A omissão dessas escolhas impede que se avalie se as inferências de validade são metodologicamente sustentadas^4^.

Quanto ao delineamento amostral, o artigo não apresenta cálculo de tamanho da amostra e adota uma amostragem por conveniência, com mais de 58% dos participantes provenientes da região Sudeste. Dado o objetivo declarado de validação nacional, a ausência de análise de invariância de medida entre regiões configura lacuna relevante. Adicionalmente, amostras superiores a 400 casos aumentam o poder estatístico e podem mascarar os efeitos da não normalidade multivariada – pressuposto que, assim como a verificação de *outliers* multivariados, nem sequer é mencionado no artigo, comprometendo a análise fatorial confirmatória (AFC) independentemente dos índices de ajuste obtidos.

A validade convergente do modelo também não foi adequadamente demonstrada. Conforme regras de bolso amplamente difundidas e exemplificadas em Hair et al.^4^, seriam necessárias, no mínimo, cargas fatoriais (λ) ≥ 0,5, variância média extraída (VME) ≥ 0,5 e confiabilidade do construto ≥ 0,7. Nenhum desses indicadores é reportado. A utilização de *network analysis* na mesma amostra da análise fatorial exploratória (AFE) e da AFC não constitui uma validação independente, mas tão somente outra perspectiva sobre os mesmos padrões de covariância^5^, não suprindo a ausência de validação cruzada em amostra independente.

Por fim, embora as medidas de confiabilidade (Alfa de Cronbach, GLB e Ômega) apresentem valores satisfatórios, confiabilidade elevada não constitui evidência de validade – distinção fundamental que o artigo não explicita.

Diante do exposto, solicitamos ao Senhor Editor: (1) a publicação desta carta como comentário ao artigo, com direito de réplica aos autores; e (2) a avaliação da pertinência de solicitar errata ou adendo que declare o estimador utilizado, os pressupostos verificados e os indicadores de validade convergente. A solidez metodológica de um instrumento com alcance nacional em saúde mental é uma responsabilidade que transcende o debate acadêmico.
