# Supplementary material for: Methodological considerations on the validation of the CuidaSM Scale
Source: Rev Saude Publica. 2026 Aug 31;60:e37. doi: 10.11606/s1518-8787.2026060007752 (PMC13645322; doi:10.11606/s1518-8787.2026060007752)
Supplement: Related texts

# Reply: Response letter: Furthering the methodological debate on the validation of the CuidaSM Scale

**Authors:** Flávio Rebustini, Ilana Eshriqui, Daiana Bonfim, Joana Moscoso Teixeira de Mendonça, Ana Alice Freire de Sousa, Letícia Yamawaka de Almeida

**Affiliations:** I Universidade de São Paulo Escola de Artes, Ciências e Humanidades Departamento de Gerontologia São Paulo SP Brasil Universidade de São Paulo. Escola de Artes, Ciências e Humanidades. Departamento de Gerontologia. São Paulo, SP, Brasil; II Hospital Israelita Albert Einstein Centro de Estudo, Pesquisa, Prática em APS e Rede São Paulo SP Brasil Hospital Israelita Albert Einstein. Centro de Estudo, Pesquisa, Prática em APS e Rede. São Paulo, SP, Brasil

We appreciate the opportunity to further the methodological debate surrounding the CuidaSM scale, broadening the discussion based on technical and scientific considerations throughout this response.

The claims that the original study on CuidaSM was a preprint without a definitive DOI and without completed peer review could reflect a transitional editorial stage, given the time lags in editorial processes. However, these claims do not apply, as the article in question, which describes in detail the development and validation of CuidaSM, has been published in PLOS One since July 10, 2025, with a definitive DOI and completed peer review^1^. Thus, it is incorrect to suggest that the evidence for CuidaSM is limited to the article published in the Revista de Saúde Pública^2^, which had a specific objective: to present additional evidence of internal structure through confirmatory factor analysis (CFA) and network analysis.

Regarding the dichotomous nature of the items, which the authors of the letter identified as a critical issue, we emphasize that this does not constitute a limitation, given that the study^1^reports the use of tetrachoric correlations, robust parallel analysis, unweighted least squares extraction, and oblique rotation—procedures compatible with binary items. The allegation of inadequacy due to the alleged use of Pearson’s correlation or an inappropriate estimator is based on inference. We would like to take this opportunity to clarify that, when selecting the estimator in Jeffreys’s Amazing Statistics Program (JASP), once the data is identified as dichotomous (data type), the system automatically applies the tetrachoric correlation and makes the necessary adjustment (see JASP’s technical notes); for AFC, the method used is DWLS. Therefore, all internal structure techniques adopted in the study used the tetrachoric correlation.

The requirement of extracted mean variance and composite reliability as mandatory criteria for accepting validity is reductionist. Standards define validity as a cumulative argument, supported by multiple sources of evidence, rather than by a single statistical index^3^. Contemporary literature emphasizes that validation studies should integrate evidence regarding content, internal structure, the response process, relationships with other variables, and consequences of use^4^. Similarly, current recommendations for factor analysis indicate that sample size and solution stability should be evaluated based on the characteristics of the factor structure itself, rather than on fixed rules or isolated indicators^5 , 6^.

Sample size calculations in psychometrics are not equivalent to those in epidemiology; there are usually minimum recommendations. Thus, the argument regarding the impact of the sample on the distribution and the impact of outliers on the analyses does not apply, as there is no assumption of a specific distribution for ordinal data. This raises a question: How can outliers be detected on a dichotomous ordinal scale? Regarding the use of invariance, there are other techniques for testing a model’s stability, including indicators described in the manuscript^1^.

Recent literature also recommends caution regarding an uncritical interpretation of AFC. Lorenzo-Seva^7^proposed mild-restricted confirmatory factor analysis to address a recurring limitation of classical CFA (unfortunately unavailable at the time of the analyses and manuscript preparation): the assumption that each item is a pure indicator of a factor, with secondary loadings, correlations between factors, or correlated residuals exactly equal to zero. This is not a point raised solely in this article; there is significant literature on these issues in AFC. In assessment instruments, particularly in health-related scales, it is common for items to be close, but not perfectly pure, indicators of a co’nstruct. When AFC artificially forces these small relationships to zero, biases may occur in the estimated parameters, leading to artificial improvements or worsening of overall indices and interpretations that are overly dependent on post hoc modifications. The combination of techniques addresses precisely the possibility of different approaches taken by the techniques, along with their strengths and weaknesses. This discussion aligns with the tradition of semiconfirmatory solutions, in which theoretical hypotheses are tested without imposing restrictions incompatible with the empirical complexity of the items^8^. Therefore, the criticism that demands the absolute centrality of AFC and a limited number of global indices runs precisely into the problem that contemporary literature warns against: that good fit indices are not, in and of themselves, a guarantee of substantive validity. For this reason, the combination of AFE, cross-validation, factor quality indicators, normalization, AFC, and network analysis should be interpreted as a strategy for integrating evidence, not as a methodological weakness. It should be emphasized that CuidaSM is part of a group of tools used worldwide that comply with recommendations for integrating techniques and accumulating evidence, having been tested using unrestricted techniques (AFE and network analysis) and a restricted technique (AFC). We would like to point out that at no point was it stated that reliability indices constitute evidence of validity.

As for the requirement to assess convergent validity, it is worth noting that this can be done in AFC/SEM using statistical techniques; however, as Sireci^4^points out, it is part of the validity evidence involving other variables. The discrepancy arises because, in AFC/SEM models, some authors use terms such as “internal convergent validity” to refer to high factor loadings, AVE/VME, and composite reliability within the same factor. However, this is a tradition more closely associated with structural equation modeling—particularly that of Fornell and Larcker—rather than with the core classification outlined in the standards^4^, which is the document we use to underpin our entire process. This reflects a narrow view of the process of seeking evidence of an instrument’s validity, which is a mixed-methods approach par excellence and has not, for decades, been confined to the limitations of any single technique.

For these reasons, we believe that the letter should not be accepted as a methodological correction to the article, as it reduces the evidence of validity to partial requirements, since it ignores the peer-reviewed publication already available, disregards the analyses described in the study, and interprets the validity criteria—as well as the set of techniques and indicators used, some of which are still in their infancy in Brazilian studies—in a restrictive manner.

Additionally, regarding the suggestion of nationwide implementation of the tool, which arose from media coverage, we believe that it goes beyond the scope proposed in the studies^1 , 2^. We acknowledge, however, that the concern regarding the incorporation of technologies into the health care system is legitimate and shared by the tool’s authors. From this perspective, the discussion can be enriched by other research approaches that foster dialogue, for example, with the field of implementation science. Thus, reinforcing our commitment to science and to strengthening the Unified Health System, we value the debate among researchers as an integral part of the knowledge-production process and remain open to continuing this dialogue.

## References

[1] 1. Mendonça JMT, Sousa AAF, Eshriqui I, Rebustini F, Bonfim D, Zorzi DS et al. Brazilian scale for evaluation of mental health care needs (CuidaSM): evidence of validity in primary health care. PLOS One. 2025;20(7):e0323833. 10.1371/journal.pone.0323833 PMC1224458440638612

[2] 2. Mendonça JMT, Rebustini F, Sousa AAF, Eshriqui I, Bonfim D, Almeida LY. Escala de avaliação da necessidade de cuidado em saúde mental: evidências adicionais. Rev Saúde Pública. 2023; 57(Supl 3):7s. 10.11606/s1518-8787.2023057005347

[3] 3. American Educational Research Association. Standards for educational and psychological testing. Washington: AERA; 2014.

[4] 4. Sireci SG, Benítez I. Evidence for test validation: a guide for practitioners. Psicothema. 2023;35(3):217-226. 10.7334/psicothema2022.477 37493145

[5] 5. Lorenzo-Seva U, Ferrando PJ. Determining sample size requirements in EFA solutions: a simple empirical proposal. Mult Behav Res. 2024;59(5):899-912. 10.1080/00273171.2024.2342324 38717588

[6] 6. Ferrando PJ, Lorenzo-Seva U, Hernández-Dorado A, Muñiz J. Decalogue for the factor analysis of test items. Psicothema. 2022;34(1):7-17. 10.7334/psicothema2021.456 35048890

[7] 7. Lorenzo-Seva U. Mild-restricted confirmatory factor analysis: a new approach to assessing population factor models. Comm Stat Simul Comput. 2025;55(5);1656-74. 10.1080/03610918.2025.2453473

[8] 8. Lorenzo-Seva U, Ferrando PJ. Factor 9.2: a comprehensive program for fitting exploratory and semiconfirmatory factor analysis and IRT models. Appl Psychol Meas. 2013;37(6):497-8. 10.1177/0146621613487794

# Reply: Carta resposta: aprofundamento do debate metodológico sobre a validação da escala CuidaSM

**Authors:** Flávio Rebustini, Ilana Eshriqui, Daiana Bonfim, Joana Moscoso Teixeira de Mendonça, Ana Alice Freire de Sousa, Letícia Yamawaka de Almeida

**Affiliations:** I Brasil Universidade de São Paulo. Escola de Artes, Ciências e Humanidades. Departamento de Gerontologia. São Paulo, SP, Brasil; II Brasil Hospital Israelita Albert Einstein. Centro de Estudo, Pesquisa, Prática em APS e Rede. São Paulo, SP, Brasil

Agradecemos a oportunidade de aprofundar o debate metodológico em torno da escala CuidaSM, ampliando a discussão a partir de considerações técnicas e científicas ao longo desta resposta.

As afirmações de que o estudo de origem da CuidaSM consistiria em um *preprint* sem DOI definitivo e sem revisão por pares concluída poderiam corresponder a uma etapa editorial transitória, considerando os descompassos temporais dos processos editoriais. No entanto, elas não se aplicam, pois o referido artigo que descreve de forma detalhada o desenvolvimento e a validação da CuidaSM encontra-se publicado na *PLOS One* desde 10 de julho de 2025, com DOI definitivo e revisão por pares concluída^1^. Assim, é equivocado sugerir que as evidências da CuidaSM se restringem ao artigo publicado na Revista de Saúde Pública^2^, o qual teve objetivo delimitado: apresentar evidências adicionais de estrutura interna por meio de análise fatorial confirmatória (AFC) e *network analysis.*

Quanto à natureza dicotômica dos itens, apontada pelos autores da carta como um ponto crítico, destacamos que não se configura como uma limitação considerando que o estudo^1^informa o uso de correlações tetracóricas, análise paralela robusta, extração por *unweighted least squares* e rotação oblíqua, procedimentos compatíveis com itens binários. A alegação de inadequação por suposto uso de correlação de Pearson ou estimador inapropriado decorre de inferência. Aproveitamos para esclarecer que, ao escolher o estimador no *Jeffreys’s Amazing Statistics* Program (JASP), ao se identificar que o dado é dicotômico (natureza do dado), a correlação aplicada é a tetracórica, o próprio sistema faz o ajuste (vide as notas técnicas do JASP), para a AFC é o DWLS. Portanto, todas as técnicas de estrutura interna adotadas no estudo utilizaram a correlação tetracórica.

A exigência de variância média extraída e confiabilidade composta como critérios obrigatórios para aceitação da validade é reducionista. Os *standards* definem validade como argumento acumulativo, sustentado por múltiplas fontes de evidência, e não por um único índice estatístico^3^. A literatura contemporânea reforça que estudos de validação devem integrar evidências de conteúdo, estrutura interna, processo de resposta, relações com outras variáveis e consequências do uso^4^. No mesmo sentido, recomendações atuais para análise fatorial indicam que o tamanho amostral e a estabilidade da solução devem ser avaliados conforme as características da própria estrutura fatorial, e não por regras fixas ou indicadores isolados^5 , 6^.

Os cálculos de amostra em psicometria não são equivalentes aos epidemiológicos; usualmente há recomendações mínimas. Assim, não se aplica o argumento do impacto da amostra sobre a distribuição e de *outliers* e seus impactos sobre as análises, pois não há pressuposto de distribuição em dados ordinais. Cabe uma pergunta: Como detectar *outliers* em uma escala ordinal dicotômica? Quanto ao uso da invariância, há outras técnicas para se testar a estabilidade de um modelo, inclusive indicadores que estão no manuscrito^1^.

A literatura recente também recomenda cautela com a leitura acrítica da AFC. Lorenzo-Seva^7^propôs a *mild-restricted confirmatory factor analysis* para enfrentar uma limitação recorrente da AFC clássica (infelizmente indisponível na época das análises e da preparação dos manuscritos): a suposição de que cada item seja um indicador puro de um fator, com cargas secundárias, correlações entre fatores ou resíduos correlacionados exatamente iguais a zero. Esse não é um apontamento apenas desse artigo; há literatura importante sobre esses problemas da AFC. Em instrumentos, sobretudo em escalas aplicadas à saúde, é frequente que os itens sejam indicadores próximos, mas não perfeitamente puros, de um construto. Quando a AFC força artificialmente essas pequenas relações a zero, podem ocorrer vieses nos parâmetros estimados, melhora ou piora artificial dos índices globais e interpretações excessivamente dependentes de modificações pós- *hoc* . A combinação de técnicas trata exatamente da possibilidade de abordagens diferentes feitas pelas técnicas, suas forças e fraquezas. Essa discussão é convergente com a tradição de soluções semiconfirmatórias, nas quais hipóteses teóricas são testadas sem impor restrições incompatíveis com a complexidade empírica dos itens^8^. Portanto, a crítica que exige centralidade absoluta da AFC e de poucos índices globais incorre justamente no problema que a literatura contemporânea alerta que bons índices de ajuste não são, por si sós, garantia de validade substantiva. Por essa razão, a combinação de AFE, validação cruzada, indicadores de qualidade fatorial, normalização, AFC e *network analysis* deve ser interpretada como estratégia de integração de evidências, não como fragilidade metodológica. Reforça-se que a CuidaSM faz parte de um grupo de instrumentos no mundo que atendem às recomendações de integração de técnicas e de acúmulo de evidências, tendo sido testado com técnicas irrestritas (AFE e *network analysis)* e técnica restrita (AFC). Oportunamente, ressaltamos que em nenhum trecho foi afirmado que os índices de confiabilidade constituem evidências de validade.

Quanto à exigência de realizarmos a validade convergente, é interessante que ela possa ser feita na AFC/SEM com uso de técnica estatística, contudo, como aponta Sireci^4^, ela está nas evidências de validade com outras variáveis. O descompasso acontece porque, em modelos de AFC/SEM, alguns autores usam expressões como “validade convergente interna” para se referir a cargas fatoriais altas, AVE/VME e confiabilidade composta dentro de um mesmo fator. No entanto, isso é uma tradição mais associada à modelagem de equações estruturais, especialmente à de Fornell e Larcker, e não à classificação central dos *standards* ^4^, que é o documento que utilizamos para fundamentar todo o nosso processo. Isso demonstra uma visão reduzida do que é o processo de busca de evidências de validade de um instrumento, que é um método misto por excelência e não se coaduna, há décadas, com a limitação de uma ou outra técnica.

Por essas razões, entendemos que a carta não deve ser acolhida como correção metodológica do artigo, pois reduz as evidências de validade a exigências parciais, uma vez que ignora a publicação revisada por pares já disponível, desconsidera as análises descritas no estudo e interpreta de forma restritiva os critérios de validade, bem como o conjunto de técnicas e indicadores utilizados, entre os quais alguns ainda são incipientes em estudos no Brasil.

Adicionalmente, em relação ao apontamento da implantação nacional da ferramenta, advindo de circulação em mídia, entendemos que extrapola o escopo proposto nos estudos^1 , 2^. Reconhece-se, entretanto, que a preocupação com a incorporação de tecnologias no sistema de saúde é legítima e compartilhada pelos autores da ferramenta. Nessa perspectiva, a discussão pode ser enriquecida com outras abordagens de investigações que avançam para o diálogo, por exemplo, com o campo da ciência da implementação. Assim, reforçando o compromisso com a ciência e com o fortalecimento do Sistema Único de Saúde, valorizamos o debate entre os pesquisadores como parte integrante do processo de produção do conhecimento e permanecemos abertos à continuidade desta interlocução.
